# Spatial transmission and meteorological determinants of tuberculosis incidence in Qinghai Province, China: a spatial clustering panel analysis

**DOI:** 10.1186/s40249-016-0139-4

**Published:** 2016-06-02

**Authors:** Hua-Xiang Rao, Xi Zhang, Lei Zhao, Juan Yu, Wen Ren, Xue-Lei Zhang, Yong-Cheng Ma, Yan Shi, Bin-Zhong Ma, Xiang Wang, Zhen Wei, Hua-Fang Wang, Li-Xia Qiu

**Affiliations:** Department of Health Statistics, School of Public Health, Shanxi Medical University, No.56 Xinjian South Road, Taiyuan, Shanxi 030001 China; Department of Epidemiology, Richard M. Fairbanks School of Public Health, Indiana University, Indianapolis, IN 46202 USA; Department of Biochemistry and Molecular Biology, School of Basic Medical Sciences, Shanxi Medical University, Taiyuan, Shanxi 030001 China; Institute for Communicable Disease Control and Prevention, Qinghai Center for Disease Control and Prevention, Xining, Qinghai 810007 China

**Keywords:** Tuberculosis incidence, Meteorological factors, Spatial clustering, Spatial panel data model

## Abstract

**Background:**

Tuberculosis (TB) is the notifiable infectious disease with the second highest incidence in the Qinghai province, a province with poor primary health care infrastructure. Understanding the spatial distribution of TB and related environmental factors is necessary for developing effective strategies to control and further eliminate TB.

**Methods:**

Our TB incidence data and meteorological data were extracted from the China Information System of Disease Control and Prevention and statistical yearbooks, respectively. We calculated the global and local Moran’s *I* by using spatial autocorrelation analysis to detect the spatial clustering of TB incidence each year. A spatial panel data model was applied to examine the associations of meteorological factors with TB incidence after adjustment of spatial individual effects and spatial autocorrelation.

**Results:**

The Local Moran’s *I* method detected 11 counties with a significantly high-high spatial clustering (average annual incidence: 294/100 000) and 17 counties with a significantly low-low spatial clustering (average annual incidence: 68/100 000) of TB annual incidence within the examined five-year period; the global Moran’s *I* values ranged from 0.40 to 0.58 (all *P*-values < 0.05). The TB incidence was positively associated with the temperature, precipitation, and wind speed (all *P*-values < 0.05), which were confirmed by the spatial panel data model. Each 10 °C, 2 cm, and 1 m/s increase in temperature, precipitation, and wind speed associated with 9 % and 3 % decrements and a 7 % increment in the TB incidence, respectively.

**Conclusions:**

High TB incidence areas were mainly concentrated in south-western Qinghai, while low TB incidence areas clustered in eastern and north-western Qinghai. Areas with low temperature and precipitation and with strong wind speeds tended to have higher TB incidences.

**Electronic supplementary material:**

The online version of this article (doi:10.1186/s40249-016-0139-4) contains supplementary material, which is available to authorized users.

## Multilingual abstracts

Please see Additional file 1 for translations of the abstract into the six official working languages of the United Nations.

## Background

Tuberculosis (TB) remains a major public health burden in many developing countries [1–3]. According to the World Health Organization TB annual report, in 2013, the number of new reported TB cases in the world was estimated at 11.4 million. Among the 22 high TB burden countries and regions, mainland China ranks second, with 1.3 million cases, giving an incidence of 98/100 000 [4]. Based on the data from the fifth TB epidemiological sampling survey in China, the TB number was higher in the western regions compared with the central and eastern regions [5]. The occurrence of TB in China has obvious periodic and seasonal features, more frequently occurring in the winter and spring, which suggests that TB might be associated with meteorological factors [6]. Current evidence has suggested that, besides the traditional factors such as genetic susceptibility [7], sex [8], education, ethnicity, drinking [9], smoking [10], and related diseases, several ecological factors, including geographic, climatic, and socioeconomic factors, also have critical impacts on the prevalence of TB [11–13].

Understanding the spatial variations in TB prevalence and its determinants is crucial for improved targeting of interventions and resources. Many geospatial analytical methods, such as spatial autocorrelation analysis (Moran’s *I* and Getis-Ord *G*) [14–16] and space-time scan statistic (SaTScan) methods [17–19] have been used for understanding TB and other public health problems [20, 21]. In China, county-level studies have used various spatial epidemiological methods to identify clustering of health conditions, including notifiable pandemic influenza A in Hong Kong [22], and TB in Linyi [2], Beijing [5], and Xinjiang [23]. However, there are currently no related studies on geospatial distribution of TB in Qinghai province.

Moreover, while several studies have shown that the TB incidence might be related with the temperature, precipitation, and wind speed [24], few studies have considered the modifier effects from time and spatial factors in the relationship between meteorological factors and TB incidence. With further research of spatial econometrics and the rapid development of computer technology, spatial autocorrelation and spatial panel data models are becoming useful tools in the analysis of spatiotemporal data, and are gradually being applied to the research of infectious diseases [24–26]. Therefore, in this study, we aimed to understand the distribution of TB and to explore the associations between meteorological factors and TB incidence using spatial autocorrelation analysis and a spatial panel data model based on surveillance data from the Qinghai Center for Disease Control and Prevention.

## Methods

**Fig. 1:**
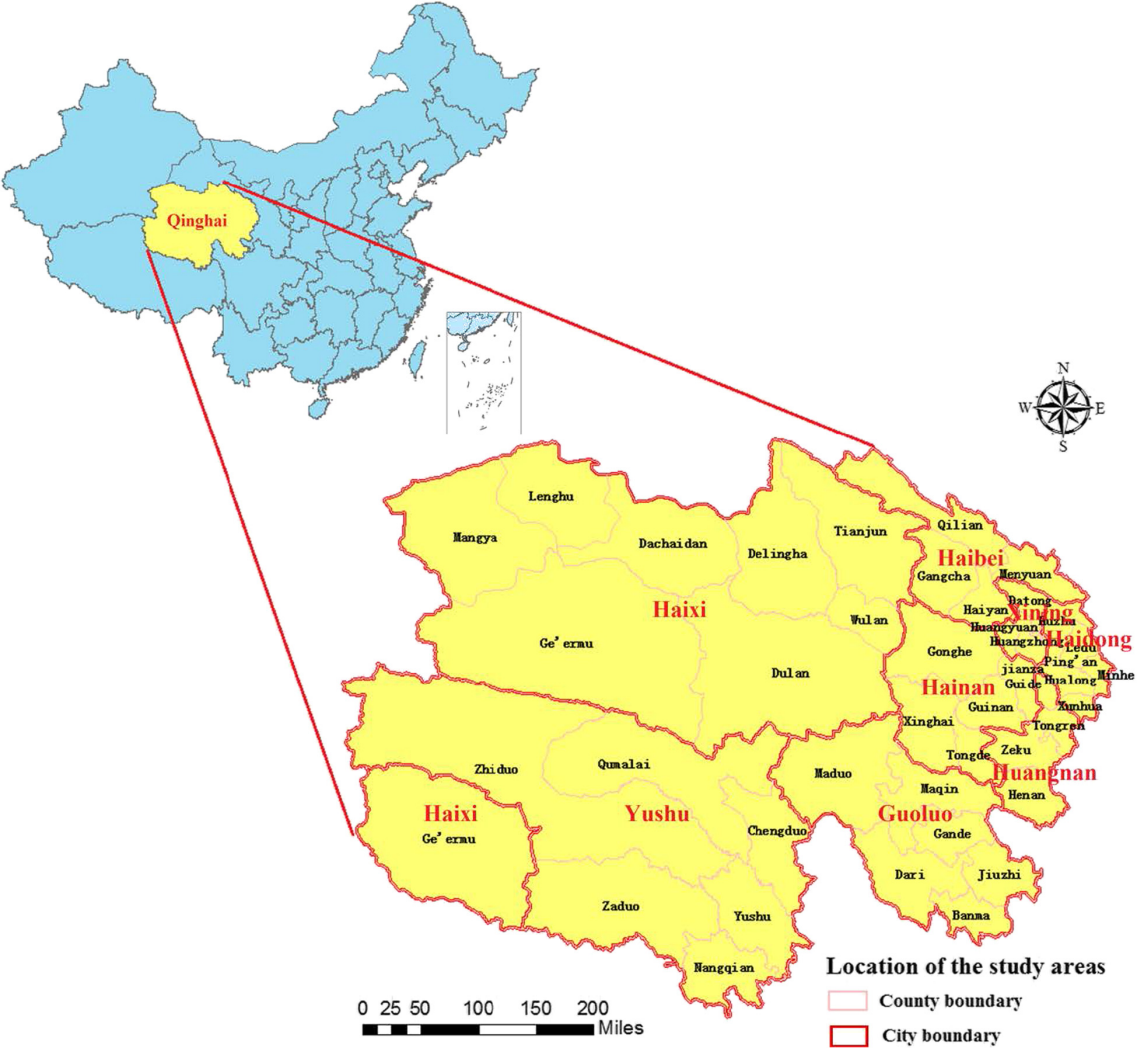
Location of the study areas, Qinghai Province, China. The map was created using the ArcGIS software (version 10.0, ESRI Inc., Redlands, CA, USA)

Qinghai province, which comprises 8 cities, including a total of 46 counties, is located between longitude 89°35′ and 103°04′ East, and latitude 31°40′ and 39°19′ North (Fig. 1). As an underdeveloped region in north-western China, it has a higher annual incidence of TB than other regions of the country. The average altitude is 3 000-5 000 m, with a typical plateau continental climate, which includes little rain, low temperatures, and long sunshine hours.

### Tuberculosis incidence data

In this study, we focused on the cases of pulmonary TB. Our TB data were based on the China Information System for Disease Control and Prevention (CISDCP, http://1.202.129.170/UVSSERVER2.0), which was established in 2005. TB cases were diagnosed using X-ray, pathogen detection, and pathologic diagnosis according to the diagnosis criteria recommended by the National Health and Family Planning Commission of the People’s Republic of China (Former Ministry of Health) in 2008 [6]. The relevant information, including age, sex, occupation, diagnostic category, and diagnostic date, was collected to analyse the epidemic characteristics of TB in Qinghai province.

TB is a notifiable infectious disease in China; all cases must be reported online within 24 h after diagnosis in the hospital. We collected the county- and city-level data from January 2009 to December 2013 in Qinghai and randomly selected 261 of all 771 medical institutions in Qinghai province and checked all medical records of these selected institutions to confirm whether there were missing TB cases or not. During the period from 2009 to 2013, in all 46 counties, a total of 27 665 TB cases and 51 TB-related deaths were identified; no missing cases or outbreaks were declared.

### Environmental data

Our meteorological data were based on the statistical data from the Qinghai statistics office, which is reported yearly by the meteorological bureau of each city. In this study, we focused on four main meteorological factors, including the monthly average temperature (MAT, °C), precipitation (MP, mm), total sunshine hours (MSH, hours), and wind speed (MAWS, m/s). Considering a time lag between infectious disease development and meteorological factors, the meteorological data were collected from July 2008 to December 2013.

### Statistical methods

#### Spatial autocorrelation analysis method

Spatial autocorrelation analysis was conducted by using Open GeoDa software (GeoDa Center for Geospatial Analysis and Computation, Arizona State University, AZ, USA) and used to identify the spatial clustering of the annual TB incidence of all 46 counties [2, 5, 27, 28]. The row standardized first-order contiguity Rook neighbours were used as the criterion for identifying neighbours in this paper. In this rule, if regions *i* and *j* are neighbours and share a boundary, *w*_*ij*_ = 1; otherwise, *w*_*ij*_ = 0. Global Moran’s *I* was calculated to test the spatial autocorrelation of all counties in Qinghai province, ranging from -1 to +1 [5]. Positive/negative spatial autocorrelation occurs when Moran’s *I* is close to +1/-1, which indicates that areas with similar (high-high or low-low)/dissimilar (high-low or low-high) incidence of TB are clustered together [29]. Monte Carlo randomization (9999 permutations) was employed to assess the significance of Moran’s *I*, with the null hypothesis being that the distribution of TB incidence in Qinghai province is completely spatially random [27]; in other words, that the counties with high and low TB incidence are randomly distributed across the study area [30]. If the test is significant (*P* ≤ 0.05), this suggests a clustering/dispersing of the TB incidence [2, 16]. Subsequently, we used local indicators of spatial association (LISA; Local Moran’ *I*) analysis and a Moran scatter plot to examine the spatial autocorrelation of each county in Qinghai province and to determine the locations of the clusters [31]. Moran’s plot shows high-high and low-low clustering in the upper right and lower left quadrants, respectively. Statistically significant high-high, low-low, and outlier local clusters (high-low and low-high) were visualized using a cluster map with county boundaries [15].

#### Spatial panel analysis method

In our study, we used data of TB incidence and meteorological factors, which were collected from different cities monthly, to explore the associations of meteorological factors with TB incidence. The data were collected at different times (monthly) and in different areas (cities), and were hence considered repeated observations data, referred to as panel data (also known as pooled time series and cross-section data). The spatial heterogeneity and spatial dependence in different cities need to be considered in the analysis [5, 32]. A spatial panel data model is able to address data with spatial dependence and also enables researchers to consider spatial heterogeneity, which typically refers to data containing continuous observations of a number of spatial units [24–26]. Compared to traditional methods based on cross-sectional or time series models, spatial panel data models are more informative and contain more variation and less collinearity among the variables [26, 33]. The use of panel data results in a greater availability of degrees of freedom, and hence increases the efficiency of the estimation [26, 34]. Therefore, we used a spatial panel data model to examine the association between the TB incidence and meteorological factors after adjustment for the spatial confounders. As the distribution of the TB incidence rate is highly skewed, log transformation of the TB incidence was used in the analyses, according to the following formula: log incidence = lg (TB incidence). The unit root test and co-integration test were conducted to confirm the stationary of the data [35]. The spatial panel data model analyses were conducted by using Matlab R2009a (Mathworks Inc., Natick, MA, USA), and the significant level was 0.05.

## Results

**Fig. 2:**
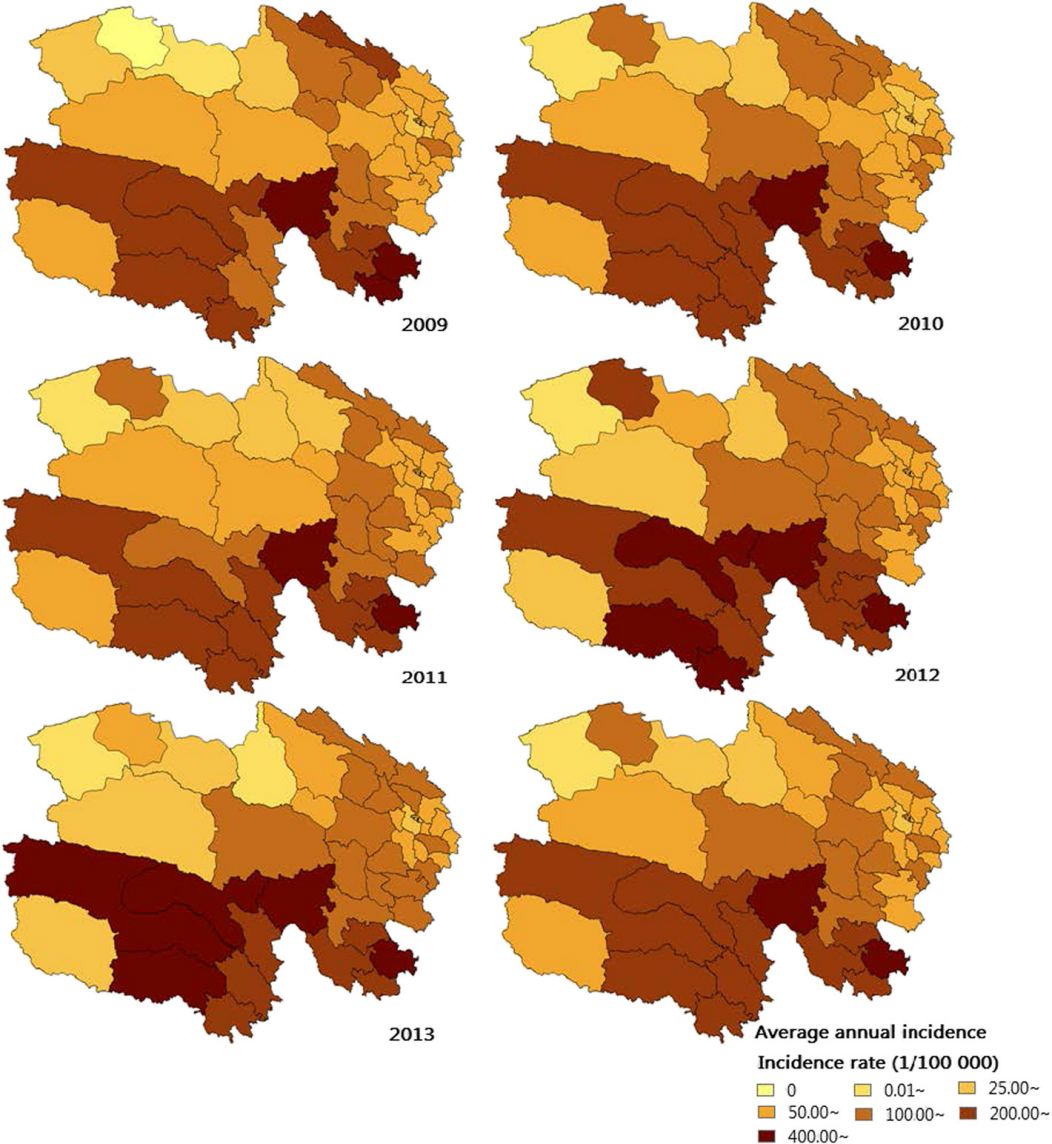
Annual incidence of tuberculosis in Qinghai Province, China, 2009-2013. The areas of high annual incidence of TB were mainly concentrated in south-western Qinghai, with the top three counties being Maduo, Jiuzhi, and Zaduo

**Fig. 3:**
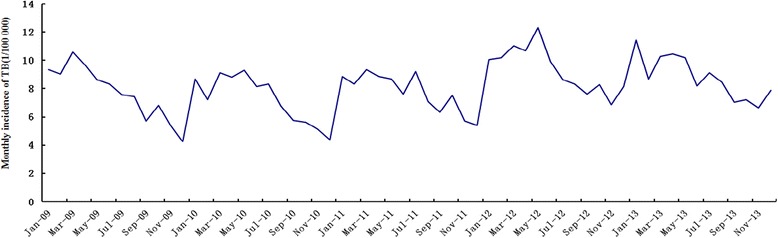
Monthly incidence rates of TB in Qinghai Province, China, from January 2009 to December 2013. The TB incidence rate showed significant periodicity and seasonality, reaching a seasonal peak around April and decreasing to a trough in December. TB, tuberculosis

Annually, the incidences of TB in Qinghai province were 93, 87, 93, 112, and 106 per 100 000 people from 2009 to 2013, accounting for 12.24, 13.96, 17.13, 19.00, and 16.84 % of all reported infectious diseases, respectively (Table 1). The high incidence areas were mainly concentrated in the cities of Yushu and Guoluo, while the top three counties of TB annual incidence were Maduo (656/100 000), Jiuzhi (461/100 000), and Zaduo (393/100 000) in the southwest of Qinghai. The low incidence areas were mainly concentrated in the cities of Haixi and Xining, with the bottom three counties being Mangya (15/100 000), Delingha (39/100 000), and Dachaidan (42/100 000) in the northwest of Qinghai (Fig. 2). The TB incidence rate showed significant periodicity and seasonality, reaching a seasonal peak around April and then decreasing to a trough in December (Fig. 3). The significant meteorological characteristics in Qinghai province included the strong sunlight and relative low temperature throughout the year (Table 2).

**Table 1:** Characteristics of tuberculosis cases in Qinghai Province, China, 2009-2013

Variables		2009		2010		2011		2012		2013	
		Report cases (incidence rate, 1/100 000)	Death cases (mortality rate, 1/100 000)	Report cases (incidence rate, 1/100 000)	Death cases (mortality rate, 1/100 000)	Report cases (incidence rate, 1/100 000)	Death cases (mortality rate, 1/100 000)	Report cases (incidence rate, 1/100 000)	Death cases (mortality rate, 1/100 000)	Report cases (incidence rate, 1/100 000)	Death cases (mortality rate, 1/100 000)
Infectious disease		42 006 (757.82)	50 (0.90)	34 865 (625.60)	29 (0.52)	30 541 (542.79)	22 (0.39)	33 514 (589.86)	42 (0.74)	35 954 (627.28)	38 (0.66)
Pulmonary tuberculosis		5 141 (92.75)	21 (0.38)	4 868 (87.35)	6 (0.11)	5 232 (92.99)	10 (0.18)	6 369 (112.10)	7 (0.12)	6 055 (105.64)	7 (0.12)
Age (years)	0-	12 (15.57)	0 (0.00)	11 (14.15)	1 (1.29)	16 (21.07)	0 (0.00)	14 (19.57)	0 (0.00)	12 (16.99)	0 (0.00)
	1-	1 (1.32)	0 (0.00)	5 (6.58)	0 (0.00)	5 (6.73)	0 (0.00)	14 (19.07)	0 (0.00)	5 (6.94)	0 (0.00)
	2-	2 (2.62)	0 (0.00)	0 (0.00)	0 (0.00)	2 (2.70)	0 (0.00)	9 (12.37)	0 (0.00)	7 (9.87)	0 (0.00)
	3-	1 (1.30)	0 (0.00)	1 (1.32)	0 (0.00)	5 (6.72)	0 (0.00)	10 (13.76)	0 (0.00)	7 (9.93)	0 (0.00)
	4-	3 (3.83)	0 (0.00)	3 (3.90)	0 (0.00)	3 (3.99)	0 (0.00)	7 (9.53)	0 (0.00)	7 (9.85)	0 (0.00)
	5-	3 (3.76)	0 (0.00)	7 (8.95)	0 (0.00)	7 (9.14)	0 (0.00)	7 (9.48)	0 (0.00)	3 (4.20)	0 (0.00)
	6-	8 (9.89)	0 (0.00)	4 (5.04)	0 (0.00)	10 (12.88)	0 (0.00)	5 (6.68)	0 (0.00)	6 (8.28)	0 (0.00)
	7-	3 (3.66)	0 (0.00)	8 (9.95)	0 (0.00)	9 (11.44)	0 (0.00)	6 (7.69)	0 (0.00)	8 (10.22)	0 (0.00)
	8-	4 (5.10)	0 (0.00)	5 (6.11)	0 (0.00)	11 (13.75)	0 (0.00)	15 (16.25)	0 (0.00)	8 (7.34)	0 (0.00)
	9-	6 (9.49)	0 (0.00)	7 (8.90)	0 (0.00)	6 (7.69)	0 (0.00)	16 (19.60)	0 (0.00)	11 (12.73)	0 (0.00)
	10-	72 (16.16)	0 (0.00)	82 (19.68)	0 (0.00)	107 (25.96)	0 (0.00)	147 (36.70)	0 (0.00)	116 (30.73)	0 (0.00)
	15-	320 (62.73)	2 (0.39)	346 (69.38)	0 (0.00)	396 (79.19)	0 (0.00)	570 (118.71)	0 (0.00)	497 (106.16)	0 (0.00)
	20-	444 (97.99)	0 (0.00)	492 (104.25)	0 (0.00)	506 (107.63)	2 (0.43)	662 (131.56)	0 (0.00)	577 (111.56)	0 (0.00)
	25-	432 (97.20)	1 (0.23)	438 (101.70)	0 (0.00)	490 (112.60)	0 (0.00)	511 (124.20)	1 (0.24)	467 (118.75)	0 (0.00)
	30-	495 (99.50)	1 (0.20)	452 (94.60)	0 (0.00)	464 (95.63)	0 (0.00)	493 (105.84)	0 (0.00)	486 (110.15)	0 (0.00)
	35-	514 (91.63)	2 (0.36)	475 (84.59)	0 (0.00)	472 (81.85)	0 (0.00)	536 (95.05)	0 (0.00)	526 (94.52)	0 (0.00)
	40-	465 (89.57)	0 (0.00)	451 (86.40)	0 (0.00)	471 (87.35)	0 (0.00)	533 (94.34)	0 (0.00)	501 (87.92)	1 (0.18)
	45-	365 (120.16)	3 (0.99)	338 (92.96)	0 (0.00)	391 (102.58)	0 (0.00)	475 (94.71)	1 (0.20)	497 (83.64)	2 (0.34)
	50-	308 (111.30)	2 (0.72)	236 (90.25)	1 (0.38)	205 (75.55)	1 (0.37)	323 (136.33)	0 (0.00)	329 (141.60)	0 (0.00)
	55-	317 (134.66)	2 (0.85)	314 (129.21)	0 (0.00)	335 (134.05)	1 (0.40)	396 (164.15)	0 (0.00)	378 (151.86)	1 (0.40)
	60-	356 (202.39)	0 (0.00)	345 (189.88)	1 (0.55)	376 (201.76)	1 (0.54)	438 (242.60)	1 (0.55)	473 (259.34)	2 (1.10)
	65-	379 (257.16)	1 (0.68)	316 (207.94)	1 (0.66)	336 (226.41)	0 (0.00)	448 (306.43)	0 (0.00)	432 (293.55)	0 (0.00)
	70-	341 (306.56)	5 (4.50)	292 (259.21)	1 (0.89)	320 (290.80)	4 (3.64)	399 (355.38)	1 (0.89)	350 (304.40)	0 (0.00)
	75-	201 (326.43)	2 (3.25)	170 (254.85)	1 (1.50)	195 (299.84)	1 (1.54)	218 (314.61)	2 (2.89)	239 (319.44)	1 (1.34)
	80-	75 (292.16)	0 (0.00)	53 (204.76)	0 (0.00)	79 (312.09)	0 (0.00)	86 (314.02)	0 (0.00)	85 (298.13)	0 (0.00)
	85-	14 (251.57)	0 (0.00)	17 (291.75)	0 (0.00)	15 (262.97)	0 (0.00)	31 (254.86)	1 (8.50)	28 (232.00)	0 (0.00)
Sex	Men	3 179 (111.66)	7 (0.25)	2 952 (103.18)	2 (0.07)	3 123 (107.18)	7 (0.24)	3 787 (131.70)	7 (0.24)	3 547 (120.26)	3 (0.10)
	Women	1 962 (72.77)	14 (0.52)	1 916 (70.65)	4 (0.15)	2 109 (77.74)	3 (0.11)	2 582 (92.01)	0 (0.00)	2 508 (90.14)	4 (0.14)
Occupation	Farmers and herdsmen	3 441 (-)	13 (-)	3 329 (-)	5 (-)	3 558 (-)	7 (-)	4 328 (-)	6 (-)	4 249 (-)	4 (-)
	Student	354 (-)	1 (-)	383 (-)	0 (-)	475 (-)	0 (-)	685 (-)	0 (-)	592 (-)	0 (-)
	Worker	333 (-)	1 (-)	317 (-)	0 (-)	302 (-)	0 (-)	264 (-)	0 (-)	228 (-)	0 (-)
	Attendant	79 (-)	0 (-)	60 (-)	0 (-)	66 (-)	0 (-)	59 (-)	0 (-)	36 (-)	0 (-)
	Teacher	60 (-)	1 (-)	34 (-)	0 (-)	36 (-)	0 (-)	39 (-)	0 (-)	30 (-)	0 (-)
	Medical personnel	19 (-)	0 (-)	16 (-)	0 (-)	29 (-)	0 (-)	29 (-)	0 (-)	23 (-)	0 (-)
	Unemployed and retirees	855 (-)	5 (-)	729 (-)	1 (-)	766 (-)	3 (-)	965 (-)	1 (-)	897 (-)	3 (-)
Diagnostic category	Sputum smear positive	2 710 (-)	12 (-)	2 633 (-)	5 (-)	2 670 (-)	5 (-)	2 665 (-)	6 (-)	2 326 (-)	5 (-)
	Bacterium negative	1 431 (-)	6 (-)	1 328 (-)	1 (-)	1 582 (-)	4 (-)	2 164 (-)	0 (-)	2 374 (-)	2 (-)
	No detection in sputum	974 (-)	3 (-)	900 (-)	0 (-)	958 (-)	1 (-)	1 528 (-)	1 (-)	1 333 (-)	0 (-)
	Only germiculture positive	26 (-)	0 (-)	7 (-)	0 (-)	22 (-)	0 (-)	12 (-)	0 (-)	22 (-)	0 (-)

**Table 2:** Descriptive statistics for meteorological variables in Qinghai Province, China, from July 2008 to December 2013

District, city	Meteorological variables	Mean	Standard deviation	Minimum	Maximum
Total	MAT (°C)	5.0	8.9	-13.4	20.4
	MP (mm)	35.3	39.4	0.0	195.1
	MSH (hours)	218.9	31.8	99.6	307.9
	MAWS (m/s)	1.5	0.5	0.1	3.5
Xining	MAT (°C)	6.1	9.1	-11.2	18.8
	MP (mm)	35.4	36.8	0.0	147.7
	MSH (hours)	217.7	29.5	151.9	289.6
	MAWS (m/s)	1.0	0.2	0.7	1.8
Haidong	MAT (°C)	7.8	9.1	-9.9	20.4
	MP (mm)	28.6	30.4	0.0	128.9
	MSH (hours)	222.2	27.9	156.6	282.2
	MAWS (m/s)	2.0	0.4	1.0	2.6
Hainan	MAT (°C)	5.6	8.8	-9.8	18.8
	MP (mm)	28.0	31.3	0.0	101.4
	MSH (hours)	238.3	28.1	176.6	303.8
	MAWS (m/s)	1.6	0.5	0.7	3.5
Haibei	MAT (°C)	2.1	9.1	-13.4	14.9
	MP (mm)	41.0	42.7	0.0	195.1
	MSH (hours)	206.2	24.4	133.1	262.3
	MAWS (m/s)	1.5	0.4	0.6	2.5
Haixi	MAT (°C)	5.0	9.7	-11.8	19.5
	MP (mm)	21.0	30.0	0.0	124.2
	MSH (hours)	243.3	28.1	192.1	307.9
	MAWS (m/s)	1.6	0.5	0.9	2.6
Huangnan	MAT (°C)	7.1	8.3	-8.7	19.2
	MP (mm)	36.1	37.7	0.0	144.2
	MSH (hours)	209.7	30.0	121.3	278.6
	MAWS (m/s)	1.1	0.3	0.1	1.6
Guoluo	MAT (°C)	0.9	7.8	-12.8	12.4
	MP (mm)	47.3	50.7	0.0	169.5
	MSH (hours)	210.8	34.6	99.6	285.5
	MAWS (m/s)	1.8	0.4	1.2	2.7
Yushu	MAT (°C)	5.1	7.8	-7.7	19.2
	MP (mm)	44.9	44.8	0.0	159.8
	MSH (hours)	202.9	26.3	132.9	274.1
	MAWS (m/s)	1.3	0.4	0.8	2.5

*MAT* monthly average temperature, *MP* monthly precipitation, *MSH* monthly total sunshine hours, *MAWS* monthly average wind speed

### Spatial autocorrelation analysis of TB incidence

**Fig. 4:**
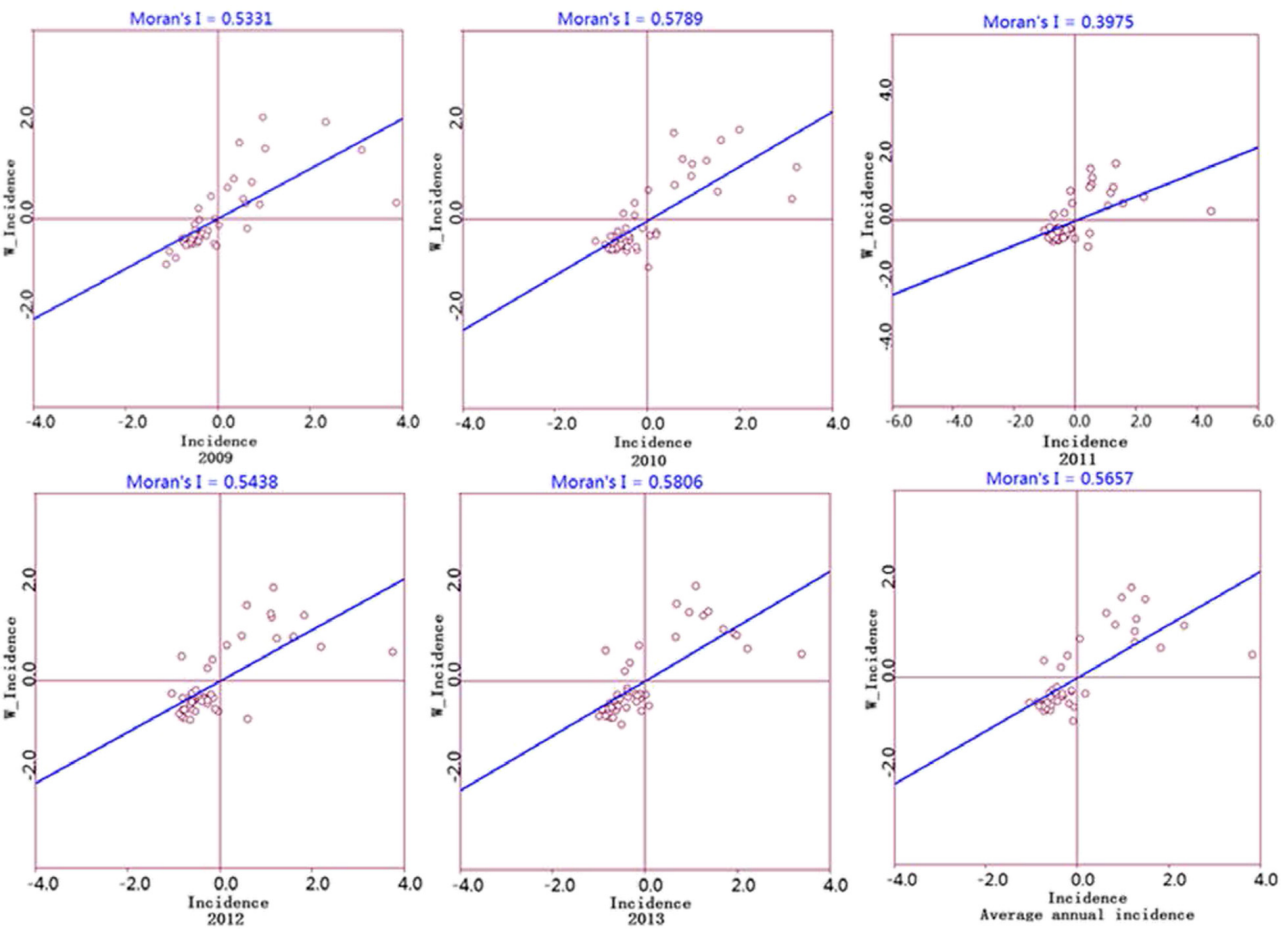
Moran scatter plot for the annual incidence of tuberculosis in Qinghai Province, China, 2009-2013. The horizontal axis shows the standardized incidence of the counties, and the vertical axis indicates the spatial lag factors; the linear slope is the Moran’s *I*

**Fig. 5:**
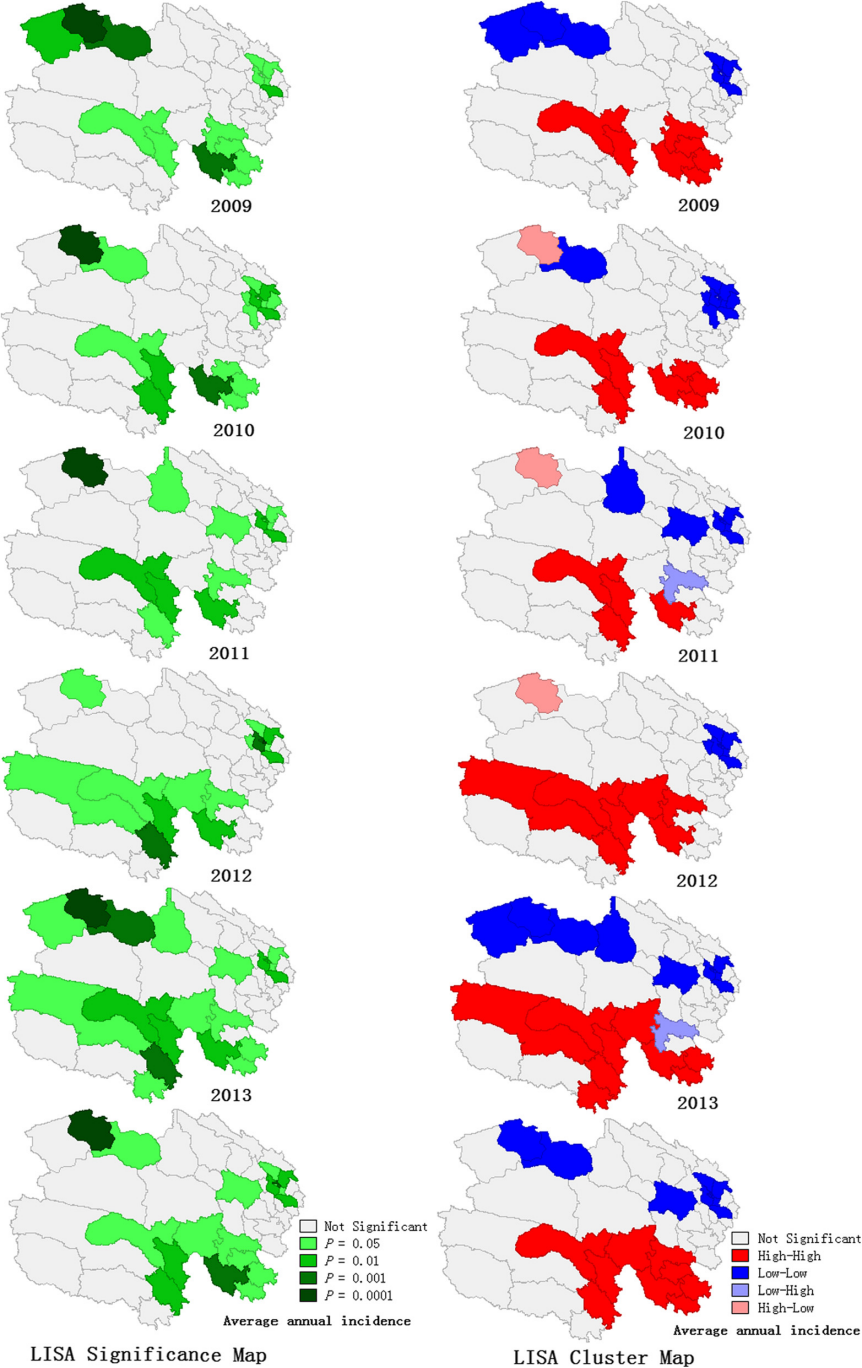
LISA significance map and cluster map for annual tuberculosis incidence in Qinghai Province, China, 2009-2013. The high risk areas were mainly concentrated in the cities of Yushu and Guoluo, while the low incidence districts were mainly distributed in the cities of Xining and Haixi. LISA, local indicators of spatial association

The global Moran’s *I* values of each year at the county level were high, ranging from 0.40 to 0.58 (all *P-*values < 0.05), which indicated that the counties with high TB incidence tended to be adjacent to the districts with high TB incidence, and that the counties with low TB incidence tended to be adjacent to the districts with low TB incidence (Fig. 4). LISA analysis revealed 11 counties with a significantly high-high spatial clustering and 17 counties with a significantly low-low spatial clustering of TB annual incidence in the five-year period. The high-high clustering areas were mainly concentrated around the cities of Yushu and Guoluo, such as Chengduo, Maduo, Qumalai, and Dari counties, with an average annual incidence of 294/100 000. The low-low clustering areas were concentrated in Xining city and surrounding areas, as well as in several counties of Haixi city, such as Mangya, Lenghu, and Dachaidan, with an average incidence of 68/100 000 (Fig. 5).

### Spatial panel analysis of meteorological factors

TB is a chronic infectious disease with a certain amount of time lag between the influencing factors and the disease. We used the meteorological factors with a 0- to 6-month lag from the incidence of TB to fit the simple linear model and classical panel data models. The associations between TB incidence and meteorological factors with a 3-month lag were found to have the best goodness of fit. Subsequently, we examined the individual effects of spatial cities by using the *Hausman*-test and *F*-test; as a result, a significant fixed effect of each city was found (*P* < 0.001). The Durbin-Watson statistic and Moran’s *I* (*P* < 0.001) indicated a spatial autocorrelation in error term. The Lagrange multiplier (LM) test showed that the spatial lag effect was more significant than the spatial error effect (Table 3). Therefore, we finally used the spatial lag fixed effects panel data model to examine the associations between TB incidence and meteorological factors.

**Table 3:** Results of the classical panel data models for the log of TB incidence with meteorological factors of a 3-month lag

Factors	Simple linear regression			Fixed effects model			Random effects model		
	Coefficient	*t*-value	*P*-value	Coefficient	*t*-value	*P*-value	Coefficient	*t*-value	*P*-value
Constant	1.0097	10.42	<0.0001				1.0367	12.27	<0.0001
MAT (°C)	-0.0195	-10.03	<0.0001	-0.0059	-4.96	<0.0001	-0.0060	-5.12	<0.0001
MP (mm)	0.0024	5.07	<0.0001	-0.0008	-2.77	0.0060	-0.0007	-2.63	0.0090
MSH (hours)	-0.0011	-2.56	0.0110	-0.0003	-1.10	0.2720	-0.0003	-1.14	0.2570
MAWS (m/s)	0.1441	5.94	<0.0001	0.0320	1.93	0.0550	0.0339	2.05	0.0410
Log likelihood	1.92			328.57					
AIC	0.01			-1.32					
SC	0.06			-1.21					
F-statistic	193.90		<0.0001						
H-statistic							10.41		0.0340
LM lag	14.14		<0.0001	39.40		<0.0001			
Robust LM lag	161.22		<0.0001	21.00		<0.0001			
LM error	34.13		<0.0001	35.93		<0.0001			
Robust LM error	181.21		<0.0001	17.54		<0.0001			
Moran’s *I*	0.20	6.07	<0.0001	0.20	6.20	<0.0001			

*TB* tuberculosis, *MAT* monthly average temperature, *MP* monthly precipitation, *MSH* monthly total sunshine hours, *MAWS* monthly average wind speed, *AIC* Akaike information criterion, *SC* Schwarz Criterion, *H-statistic* Hausman-statistic, *LM* Lagrange multiplier

The result showed that the background incidence of each city was different; the highest and lowest background incidences were found in Guoluo city (12.88/100 000) and Haixi city (2.75/100 000), respectively (Table 4). The spatial autocorrelation coefficient was 0.30, meaning that a spatial spillover phenomenon existed; the TB incidence of the study city increased 1 time when the TB incidence of adjacent cities increased 9 times and other influencing factors were kept constant. The regression coefficients of MAT, MP, and MAWS were all statistically significant (all *P*-values < 0.05), with each 10 °C, 2 cm, and 1 m/s increase in temperature, precipitation, and wind speed being associated with 9 % and 3 % decrements and a 7 % increment in the TB incidence, respectively (Table 5).

**Table 4:** Results for spatial individual effects of each city by using the spatial panel data model

Cities	Intercept term (*μ*_*i*_)	Background incidence (1/100 000)	Cities	Intercept term (*μ*_*i*_)	Background incidence (1/100 000)
Haixi	0.44	2.75	Hainan	0.70	5.01
Xining	0.51	3.24	Haibei	0.79	6.17
Haidong	0.57	3.72	Yushu	1.09	12.30
Huangnan	0.64	4.37	Guoluo	1.11	12.88

**Table 5:** Results of the spatial panel data model for the log of TB incidence with meteorological factors of a 3-month lag

Factors	Coefficient	95 % CIs of coefficients	*P*-value
MAT (°C)	-0.0040	-0.0063, -0.0017	<0.0001
MP (mm)	-0.0006	-0.0011, -0.0001	0.0250
MSH (hours)	-0.0002	-0.0007, 0.0002	0.2680
MAWS (m/s)	0.0309	0.0001, 0.0618	0.0490
*ρ*	0.2997	0.2003, 0.3992	<0.0001

*TB* tuberculosis, *CI* confidence interval, *MAT* monthly average temperature, *MP* monthly precipitation, *MSH* monthly total sunshine hours, *MAWS* monthly average wind speed, *ρ* spatial autocorrelation coefficient

## Discussion

In our study, we used the data of TB from the CISDCP to analyse the characteristics of the TB incidence in Qinghai province. The incidence of TB in Qinghai province showed clear periodic and seasonal features in the colder winter and spring months, similar to the patterns of epidemic regularity reported in Beijing [5] and Shandong [2], China, and in India [36, 37] and Mongolia [38]. In our study, it was shown that the TB incidence was higher in elders than in young people, with farmers and herdsmen at particularly high risks (Table 1), which supported the notion of TB pathogenesis regularity [39]. Moreover, the TB incidence in regions with relatively poor economic conditions and high altitudes has been shown to be high, potentially owning to differences in the ethnicity, medical and health conditions, and economic and educational levels of the residents. Additionally, the local environment and climatic conditions may also influence the incidence of TB [37, 40–43]. Thus, in Qinghai, the farmers and herdsmen living in the regions with cold climate and high altitudes should be made aware of the high risk of TB occurrence.

The incidence and spatial autocorrelation differ, but are associated in some regards. A higher incidence reflects higher epidemic strength. Spatial autocorrelation describes the relationship of incidence between one region and the surrounding areas and is based on incidence and overall consideration of regional geographical, human, and environmental factors. In our study, we chose Moran’s *I* to analyse the spatial autocorrelation. It showed a positive correlation within regions, and the correlation displayed an increasing tendency year by year, indicating that the distribution of TB in Qinghai is not random, with obvious spatial clusters. These results are consistent with previously published studies [2, 11, 37, 40].

Spatial clustering analysis has suggested that classical multiple linear regression analysis is not suitable for exploration of TB risk factors at the ecological level. Thus, this method should be combined with a spatial statistical model to explore the risk factors [25]. A panel data model can be directly used to assess the differences between regions, control for individual heterogeneity, and present more reasonable results. However, this model does not consider the spatial autocorrelation in the study area. Accordingly, a spatial panel data model combining spatial metrology with the panel data model was used in our study. This model takes the individual effects and spatial autocorrelation into consideration, which could result in better use of the spatiotemporal information from infectious diseases surveillance data [24, 26]. In a spatial panel data model, the introduction of a spatial individual effect can correct deviations caused by unobserved variables, and the use of a spatial weight matrix can illustrate the spatial correlation and reflect the interaction between regions [34]. Moreover, the modelling estimation is more effective and could help us research unknown variables in depth.

Some studies have reported that the average incubation period of TB ranges from four to eight weeks, with a two-month interval from the symptom appearance to medical diagnosis [6]. Accordingly, we created a fitting model with a 3-month lag [44–46]. The results showed that the combined data using the traditional method would overstate the influence of the meteorological factors, because the effect is different in different regions. Conversely, the spatial panel data model reduced error occurrence and increased the goodness of fit. The spatial autocorrelation coefficient was 0.30, which indicated that the introduction of the spatial lag dependent variable could reasonably explain the spatial autocorrelation. Further, the regression coefficients of MAT and MP were negative, while that of MAWS was positive, suggesting that with increasing MAT and MP, the incidence of TB decreases exponentially. Conversely, with increasing MAWS, the incidence increases exponentially. These result are in accordance with the findings of previous studies, such as those by Li et al. [6] and Naranbat et al. [38].

In cold condition, especially in the winter, most people stay indoors for a long time, hence, once someone releases bacteria into the environment, elderly and immunocompromised populations are at particularly high risk of infection because of the poor ventilation. Moreover, some studies have shown a relationship between vitamin D levels and TB incidence [38]. Fewer outdoor activities and less exposure time to sunlight result in decreased synthesis of vitamin D in the human body. In turn, this may increase the risk of TB infection [6, 47]. In the traditional view, TB infection is mainly caused by bacteria in the sputum. When the sputum gets dry, bacteria are expelled into the air and enter other people’s respiratory systems. With increasing rainfalls, the air humidity increases and the transmission of aerosols decreases. Hence, the risk of bacteria entering the respiratory system is decreased, as is the incidence of TB.

In summary, we found that the spread of TB in Qinghai province is not random, but rather present obvious spatial clustering. The use of spatial statistic methods may offer necessary feedback in terms of the prevalence nature and epidemic characteristics of TB in various regions, and may consequently result in public health officials providing TB control and developing novel prevention strategies. In this study, we quantified the relationship between TB and meteorological factors using a spatial panel data model on the basis of panel data for the first time. A spatial panel data model is appropriate if longitudinal data of multiple units are available and if spatial autocorrelation exists. This model has a better model fitting and provides more precise effect size estimation. Additionally, as meteorological factors obviously affect the TB incidence in Qinghai province, future strategies of TB control and prevention should consider climate variations.

However, there are some limitations in the present study that are worth mentioning. First, as it is difficult to collect meteorological data of each county, our analysis was initiated at the city level. Second, the temperature, precipitation, sunshine hours, and wind speed are not the only meteorological factors affecting TB distribution. Therefore, additional county-level factors, such as atmospheric pressure, average vapour pressure, and average relative humidity, will be taken into consideration in our future study.

## Conclusions

Our study found that high-high clustering areas of TB incidence were mainly concentrated in the southwest, while low-low clustering areas were found mainly in eastern and north-western Qinghai. The TB incidence was positively associated with the temperature, precipitation, and wind speed after adjusting for spatial heterogeneity and spatial correlation in Qinghai province.

### Ethics and consent

The Ethics Review Board of the Qinghai Center for Disease Control and Prevention was consulted, since the project used only surveillance data, confirmed that ethics approval was not required because the data did not contain any personal or health information that could be connected back to the original identifiers.

## Additional file

Additional file 1: Multilingual abstracts in the six official working languages of the United Nations. (PDF 406 kb)

## Abbreviations

LISA: local indicators of spatial association
MAT: monthly average temperature
MAWS: monthly average wind speed
MP: monthly precipitation
MSH: monthly total sunshine hours
TB: tuberculosis
