# Supplementary material for: Spatial transmission and meteorological determinants of tuberculosis incidence in Qinghai Province, China: a spatial clustering panel analysis
Source: Infect Dis Poverty. 2016 Jun 2;5:45. doi: 10.1186/s40249-016-0139-4 (PMC4890510; doi:10.1186/s40249-016-0139-4)
Supplement: Additional file 1: — Multilingual abstracts in the six official working languages of the United Nations. (PDF 406 kb) [file 40249_2016_139_MOESM1_ESM.pdf]

Translation of the abstract into the six official working languages of the United Nations

انتقال العدوى بين الأماكن وتحديد الأرصاد الجويّة لانتشار مرض السلّ في مقاطعة تشينغهاي: البيانات المزدوجة للتجمع المكاني

هواكس-أكسيانغ راو، أكسي زهانج، لاي زاو، جوان يو، وان ران، أكسو لاي زهانج، يونج-تشانغ ما، يان شي، بين-زهونج ما، أكيانغ وانغ، زان واي، هوا فانغ وانغ، لي إكسيا كيو

ملخّص

تقديم عام: يُعتبر مرض السلّ من الأمراض المعدية التي يجب التبليغ عنها وتُسجّل أعلى ثاني نسبة للإصابة بالسلّ في مقاطعة تشينغهاي، وهي منطقة تُعاني من ضعف البنية التحتية للرعاية الصحيّة الأولية. إنّ فهم انتشار مرض السلّ أي التوزيع المكاني والعوامل البيئية المحيطة به لأمر ضروري بهدف تطوير استراتيجيات فعّالة للتحكّم في انتشار المرض والقضاء عليه.

التقنيات المستخدمة: تم جمع البيانات المتعلّقة بانتشار مرض السلّ والأرصاد الجوية بالاعتماد على كلّ من نظام المعلومات الصيني لمكافحة الأمراض والوقاية منها وكتب الإحصائيات السنوية، على التوالي. قمنا باحتساب قيمة Moran’s I العالمية والمحليّة باستعمال التحليل للارتباط التلقائي المكاني (spatial autocorrelation analysis) لتحديد التجمّعات المكانيّة التي انتشر بها السلّ سنويّا، وقد اعتمدنا على نموذج البيانات المزدوجة لفحص ترابط العوامل الجوية مع انتشار مرض السلّ وذلك بعد تعديل الآثار الفردية المكانيّة والارتباط التلقائي المكاني (spatial autocorrelation)**.**

النتائج: باستعمال طريقة Moran’s I تمّ الكشف عن 11 بلد ممّن يشهدون ارتفاعا ملحوظا في التجمّعات المكانيّة (متوسّط المعدّل السنوي: 294/100 000) و17 بلد ممن يشهدون انخفاضا ملحوظا في التجمّعات المكانيّة (متوسّط المعدّل السنوي: 68/ 100 000) لانتشار مرض السلّ خلال فترة الدراسة للخمس سنوات، وتراوحت قيمة Moran’s I بين 0.40 و0.58 (قيمة P<0.05). ارتبط انتشار مرض السلّ بدرجة الحرارة والأمطار وسرعة الرياح (قيمة P<0.05) وتمّ تأكيد هذا بنموذج البيانات المزدوجة. كلّ ارتفاع في درجة الحرارة بنسبة 10 درجة مئوية ونسبة الأمطار 2 صم وسرعة الرياح 1 متر/ثانية يليه انخفاض 9% و3% وارتفاع 7% لانتشار مرض السلّ على التوالي.

الخاتمة: تمّ تسجيل نسب مرتفعة من مرض السلّ بالأساس في جنوب غرب تشينغهاي بينما ارتكزت النسب منخفضة في شرق وشمال غرب تشينغهاي. إنّ المناطق التي تشهد درجة حرارة منخفضة وأمطار متفرّقة مع رياح قويّة، هي أكثر عرضة لارتفاع نسب انتشار مرض السلّ.

Translated from English version into Arabic by Zeineb TRABELSI, through

TRANSLATORS
WITHOUT BORDERS

应用空间聚集面板分析探索青海省肺结核病空间分布规律和气象影响因素

饶华祥，张熙，赵磊，于娟，任雯，张雪雷，马永成，石燕，马斌忠，王祥，魏珍，王华芳，仇丽霞

摘要

引言：青海省是中国基础卫生保健设施相对不足的省份之一，该地区肺结核发病率在法定报告传染疾病中位居第二。了解该地区肺结核病的空间分布规律和相关环境影响因素，可以减少肺结核病的发生，并为肺结核病的防控提供有效的策略。

方法：该研究的肺结核病发病资料来源于中国疾病预防控制信息系统，气象数据来源于青海省统计年鉴。通过全局和局部Moran’s *I*空间自相关分析在县级水平探讨肺结核病年发病率的空间分布特点。同时考虑到空间异质性和空间自相关性，采用空间面板数据模型研究气象因素与肺结核病发病率之间的关联性。

结果：青海省2009-2013年间肺结核病年发病率在县级层面全局Moran’s *I*范围为0.40-0.58（$P < 0.05$）。局部Moran’s *I*空间自相关分析共发现11个有统计学差异的高-高聚集县（年平均发病率294/10万）和17个低-低聚集县（年平均发病率68/10万）。空间面板数据模型分析结果显示肺结核病发病率与月平均气温、降水量和

风速有关。气温每增加10°C，降水量增加2cm，风速增加1m/s，肺结核发病率相应降低9%、3%和增加7%。
结论：青海省肺结核病高发地区主要集中在西南部，低发地区主要集中在东部和西北部。低气温、降水量偏少和风力较大的气象条件下肺结核发病率较高。
Translated from English version into Chinese by Rao Huaxiang, Qiu Lixia

Transmission spatiale et indicateurs météorologiques de l'incidence de la tuberculose dans la province chinoise du Qinghai : une analyse par regroupement spatial

Hua-Xiang Rao, Xi Zhang, Lei Zhao, Juan Yu, Wen Ren, Xue-Lei Zhang, Yong-Cheng Ma, Yan Shi, Bin-Zhong Ma, Xiang Wang, Zhen Wei, Hua-Fang Wang and Li-Xia Qiu

Résumé

Contexte : La tuberculose est la deuxième maladie infectieuse à déclaration obligatoire par ordre d'incidence dans le Qinghai, une province disposant d'une faible infrastructure de soins de santé primaires. Comprendre la répartition spatiale de la tuberculose et des facteurs environnementaux qui lui sont liés est nécessaire pour développer des stratégies efficaces afin de lutter contre la maladie et, à terme, de l'éliminer.

Méthodes : Nos données d'incidence de la tuberculose et météorologiques ont été extraites respectivement du Système d'information pour la lutte et la prévention des maladies en Chine et des annales statistiques. Nous avons calculé l'indice *I* de Moran global et local en utilisant une analyse par autocorrélation spatiale afin de retracer la répartition spatiale de l'incidence de la tuberculose par année. Un modèle de zonage de données spatiales a été appliqué pour examiner les associations entre les indicateurs météorologiques et l'incidence de la tuberculose après ajustement tenant compte des effets spatiaux individuels et de l'autocorrélation spatiale.

Résultats : En utilisant l'indice *I* de Moran local, nous avons identifié 11 comtés présentant un regroupement spatial significativement « plus-plus » (*high-high*, incidence moyenne annuelle : 294/100 000) et 17 comtés avec un regroupement spatial significativement « moins-moins » (*low-low*, incidence moyenne annuelle : 68/100 000) de l'incidence annuelle de la tuberculose sur la période de cinq ans étudiée ; les valeurs de l'indice *I* de Moran global étaient comprises entre 0,40 et 0,58 (toutes valeurs de $P < 0{,}05$). L'incidence de la tuberculose était associée positivement à la température, aux précipitations et à la vitesse du vent (toutes valeurs de $P < 0{,}05$), ce qui a été confirmé par le modèle des données spatiales. Chaque augmentation de 10°C de la température, 2 cm des précipitations et 1 m/s de la vitesse du vent était associée à une diminution de 9 % et 3 % et une augmentation de 7 % de l'incidence de la tuberculose, respectivement.

Conclusions : Les zones à forte incidence de tuberculose étaient principalement concentrées dans la partie Sud-Ouest de la province du Qinghai, tandis que les zones à faible incidence étaient concentrées principalement au Nord-Ouest et au Nord-Est. L'incidence de la tuberculose était plus élevée, en tendance, dans les zones où les températures étaient basses, les précipitations faibles et les vents rapides.

Translated from English version into French by Suzanne Assenat, through

TRANSLATORS
WITHOUT BORDERS

Территориальное распространение и метеорологические факторы заболеваемости туберкулезом в провинции Цинхай, Китай: Анализ пространственной кластеризации

Хуас-Сянг Жао, Си Жанг, Леи Жао, Дзуан Ю, Вен Жен, Сю-Леи Жанг, Йонг-Ченг Ма, Ян Ши, Бин-Жонг Ма, Сянг Ванг, Жен Вей, Хуа-Фанг Ванг и Ли-Ся Чю

Краткое содержание

Подоплека: Туберкулез (ТБ) – подлежащее регистрации инфекционное заболевание с вторым по уровню заболеваемости в провинции Цинхай с неразвитой инфраструктурой первичной медицинской помощи. Понимание территориального распространения ТБ и связанных с ним метеорологических факторов необходимо для разработки эффективных стратегий контроля и последующего устранения ТБ.

Методы: Наши данные о вспышках ТБ и метеорологические данные были получены из Китайской информационной системы по контролю и профилактике заболеваний, а также статистических ежегодников соответственно. Мы рассчитали глобальный и локальный индекс Морана I посредством анализа пространственной атвокорреляции для определения пространственной кластеризации заболеваемости ТБ для каждого года. Модель пространственных данных использовалась для изучения связи метеорологических факторов с заболеваемостью ТБ после корректировки пространственного индивидуального воздействия и пространственной автокорреляции.

Результаты: Локальный метод Морана I выявил 11 областей со значительно высокой пространственной кластеризацией (среднегодовое распространение: 294/100 000) и 17 областей со значительно низкой пространственной кластеризацией (среднегодовое распространение: 68/100 000) ТБ в течение рассматриваемого пятилетнего периода; шлобальный индекс Морана I колебался от 0.40 до 0.58 (все *P*-величины < 0.05). Заболеваемость туберкулезом была положительно связана с температурой, осадками и скоростью ветра (все *P*-величины < 0.05), что было подтверждено моделью пространственной панели. Каждое увеличение температуры, осадков и скорости ветра на 10°C, 2 см и 1 м/с связано с 9% и 3% уменьшением и 7% увеличениемзаболеваемости туберкулезом соответственно.

Заключение: Районы с высоким уровнем заболеваемости ТБ сосредоточены главным образом на юго-западе провинции Цинхай, а области с ником уровнем заболеваемости ТБ расположены на востоке и северо-востоке провинции Цинхай. В районах с низкими температурами и небольшим количеством осадков, без сильных ветров, как правило, случаи ТБ наблюдаются чаще.

Translated from English version into Russian by Jekaterina Merkuljeva, through

TRANSLATORS
WITHOUT BORDERS

Transmisión espacial y determinantes meteorológicos de la incidencia de la tuberculosis en la provincia Qinghai, en China: Un análisis de conglomerados espacial y de datos de panel

Huax-Xiang Rao, Xi Zhang, Lei Zhao, Juan Yu, Wen Ren, Xue-Lei Zhang, Yong-Cheng Ma, Yan Shi, Bin-Zhong Ma, Xiang Wang, Zhen Wei, Hua-Fang Wang y Li-Xia Qiu

Resumen

Antecedentes: La tuberculosis (TB) es la segunda enfermedad infecciosa reportable en cuanto a incidencia en la provincia de Qinghai, una provincia con una infraestructura muy pobre para la atención médica primaria. Es necesario comprender la distribución espacial de la TB y los factores ambientales asociados para desarrollar estrategias efectivas con el fin de controlar y eliminar aún más la TB.

Métodos: Extrajimos nuestra información sobre la incidencia de TB y los datos meteorológicos del Sistema de Información de Control y Prevención de Enfermedades de China y de anuarios estadísticos, respectivamente. Calculamos el *l* de Moran global y local mediante análisis de autocorrelación espacial para detectar los conglomerados espaciales de la incidencia de TB para cada año. Se aplicó un modelo de datos de panel espacial para examinar las relaciones entre los factores meteorológicos y la incidencia de la TB luego del ajuste de efectos espaciales individuales y de la autocorrelación espacial.

Resultados: El método *l* de Moran local detectó 11 condados con conglomerados espaciales significativamente elevados (incidencia anual promedio: 294/100 000) y 17 condados con conglomerados espaciales significativamente bajos (incidencia anual promedio: 68/100 000) de incidencia anual de TB dentro del período examinado de 5 años; los valores de *l* de Moran global variaron de 0,40 a 0,58 (todos los valores $P < 0{,}05$). La incidencia de la TB se asociaba positivamente con la temperatura, precipitación y velocidad del viento (todos valores-P < 0.05), que fueron confirmados por el modelo de datos de panel espacial. Cada 10°C, 2 cm, y 1 m/s de aumento en la temperatura, precipitación, y velocidad del viento se produjeron decrementos de 9% y 3% y un incremento del 7% en la incidencia de la TB, respectivamente.

Conclusiones: Las áreas de alta incidencia de la TB se concentraron principalmente en el sudoeste de Qinghai, mientras que las áreas de baja incidencia de la TB se agruparon en las zona este y noroeste de Qinghai. Las áreas con bajas temperaturas y precipitación y mayores velocidades de viento tendían a tener una mayor incidencia de la TB.

Translated from English version into Spanish by Maria Alejandra Aguada, through

TRANSLATORS
WITHOUT BORDERS
